# Heathen Ignorance of the Healing Art

**Published:** 1888-04

**Authors:** H. M. Scudder


### FACTS REGARDING HEATHEN IGNORANCE OF THE HEALING ART.

[Abstract of a Paper by H. M. Scudder, M. D., in the Medical Missionary Journal.]

For many years I practiced medicine and surgery among the natives of India as part of my missionary work. There are no acuter intellects on the earth than theirs. In poetry, grammar, logic, mathematics, and especially in metaphysics, the Hindoos have displayed signal ability, but in the physical sciences they are only babes. Nothing will more clearly illustrate this than a few allusions to the condition of medical and surgical science and practice among them.

WHAT DO THEY KNOW OF ANATOMY?

Absolutely nothing beyond what they see on the outside of a human being. Caste renders the dead body a source of ceremonial pollution, and nothing can be more repugnant to a well-born Hindoo than dissecting. Hence his notions of the structure of the body are exceedingly fanciful. He soberly declares that the human frame is built upon a skeleton which contains the moderate number of 4,448 bones. He furthermore asserts that in consequence of this, man is obnoxious to 4,448 diseases; a disease for each bone being a logical sequence. He maps out for you the whole inner man, though he has never examined it. He affirms it to be an organism permeated

BY TEN LARGE TUBES.

He describes tubes starting from the pelvis, part of them running upwards to branch out in the trunk, and part downwards to terminate in the big toes. He describes tubes which shoot across the body, diagonally, tubes which form circles, tubes which build arches, tubes which develop into canopies, and tubes which by their convolutions constitute regal seats upon which are enthroned certain Hindoo deities. He describes tubes which end in the eyes and ears, in the tongue and in the bones. The ten principal tubes ramify into the exact number of twelve, 72,000 divisions.

WHAT DO THEY KNOW OF PHYSIOLOGY?

One statement shall suffice. In the ten chief tubes, just mentioned, play ten vital breaths or winds. One breath, moving through its tube causes man to respire; another, urging its way through its tube, forces him to hiccough, and another makes him sneeze; another enables him to bend and stretch, another leads him to wink, another produces smiles, and another elevates the chyle.

WHAT DO THEY KNOW OF PATHOLOGY IN ITS VARIOUS DEPARTMENTS?

As to the enumeration of diseases, I have already stated that there are just 4,448. As to the causes of disease, they are amusing, if not correct. If a man spits upon the hearth-fire, or into a pan of burning coals, he will get asthma. (Would that some such consideration would frighten and restrain the tobacco chewers and spitters of this country.) If a child has thin legs, a large head, and a prominent abdomen, it is because a toad has sometime fallen upon the luckless youngster; and the way to heal him is to catch a toad and confining it for three days in a new waterpot, to draw water from the pot for the daily ablution of the child. The evil eye of a stranger, the sorceries of an enemy, the incantations of a witch, or the hostility of a demon that haunts a neighboring tree or cemetery, are considered efficacious causes of disease. If a mother has a fine, fat babe, she fears even the incautious laudatory remark of a friend. "Your babe is plump and pretty, and eats well," says an admiring female acquaintance. Alas! some malady will fall upon the infant, and to avert it, the troubled mother heats an iron and plunges it hissing into water, with which water she afterward washes the child's face. The Hindoo physician, when no other assignable cause occurs to him, finds a convenient refuge in rat bites. In my dispensary, a patient gravely declared that he was suffering from a pain in his ribs, because twelve years before a naughty rat had nibbled one of his toes. In such cases I gave a decoction of sarsaparilla with iodide of potassium, and found it very successful.

WHAT DO THE HINDOOS KNOW OF THERAPEUTICS?

They, of course, know something of the effects of certain drugs, used separately, or in combination. Especially do the Mohammedans, whose Arabian antecedents are in their favor, concoct some good medicines. Moreover, it must be allowed that the Hindoos with their remedies can treat such a disease as "the country sore eye," a form of ophthalmia peculiar to that part of the world, as well as we can with our Western methods; but their utter ignorance of anatomy, physiology, and pathology, and their feeble power of diagnosis, render, in almost all cases, their application of remedies a hit-and-miss practice, in which the missing is the law, and the hitting is the exception. They are zealous dabblers in drugs, inveterate dosers. This is to be expected. The less the man knows, the longer will be the list of his remedies, the more complicated and heroic will be his prescriptions.

Hindoo medical science leans strongly upon magical incantations. A Brahmin once came to me with an immense abscess. He had for a long time endured intolerable agony from the pressure and the tension upon certain nerves. Night after night his medical advisers had muttered their conjurations over the incorrigible swelling, of the nature of which they knew nothing. One plunge of the lancet let out his pain and a stream of pus, to the amazement and mortification of his doctors.

If a person is bitten by a dog the part is to be well slapped with an old shoe, and then treated with burned shoe-leather and the recitation of a magical formula.

If an individual is stung by a scorpion, in order to find relief he dispatches a friend to pronounce in a whisper a mystic verse in the ear of a male buffalo calf. The buffalo is a domestic animal in India. By the time the messenger finds the village herd of buffaloes, selects a male calf, catches it, constrains it to listen to his whispers, and returns, there can be no doubt that the pain of the scorpion sting is mitigated.

Their medical and surgical practice abounds in ridiculous remedies.

If a fishbone sticks fast in one's throat, help may be obtained by worshipping a black cat. Some families that are fond of fish keep black cats for that purpose.

A remedy is sometimes to be applied, first externally and then internally. In case of a local pain on the exterior of the body, one should first apply a poultice of hot rice and oil, and then after it has become cold, should swallow the poultice, thus bringing the healing agent to act on both sides of the pain (the same poultice, first outside, secondly inside). If the pain can hold out against that, it must be considered incorrigible.

Great faith is reposed, in a variety of disorders, upon the efficacy of rhinoceros horn, triturated on a stone and mixed with milk. Also peacock feathers stewed in oil are affirmed to be a soothing application for gunpowder burns. There is some poetry in this, if no curative energy, for what can be more significant of irresistible assault upon disease than the horn of a rhinoceros in the hand of a Hindoo physician, and what more suitable to grace his triumph than the brilliant plume of a peacock.

I will cite one prescription, still more strange, and which is designed to act mechanically. In case the uvula becomes elongated, and the doctor fails in reducing it to its normal state by squeezing the juice of some leaves on the crown of the head, he must select the three central hairs on the top of the head, and seizing them must suddenly and forcibly twitch them upward, in order to bring the refractory little organ into its right place.

### WHAT DO THE HINDOOS KNOW OF SURGERY?

Less than of medicine. Totally ignorant of anatomy, what can they do? The barber sometimes screws his courage up to the point of experimenting on a boil with his razor, while his coadjutor, the potter—for these two, the barber and the potter, are the surgeons—can do up a broken limb in rude splints, which he binds so tightly as often to maim the member altogether. The Musselman, belonging to a fierce race, ventures to make a plunge in the eye at an opaque lens. This is done thus: The person with cataract is made to stand. The

Musselman, armed with a shoemaker's awl, takes position in front of his victim, and tells him to look up. As the eyeball moves up, he dashes the awl into it, just below the cornea. This barbarous operation sometimes succeeds, and generally does not.

The deliberate villainy of native practitioners is astounding. The main object with most of them is to extort money, and they play in a thousand ways upon the fears and credulities of those who employ them. They are full of lies and chicaneries of every sort. A girl was brought to me who was suffering from an alveolar abscess, caused by a bad tooth. There was an external opening through the cheek. Her friends told me that the native physician declared that the disease was caused by the existence of a number of dead flies in the centre of her head, which must be extracted through the ear. He therefore visited her periodically, and working at her ear, and giving her sufficient pain to make the extraction of a fly appear a marvelous operation, he produced the insect, which he held concealed in his hand. I have forgotten how many flies he thus took out of her head; there were several. For each of them he made her parents pay handsomely.

Imagine the physical miseries which are the fruits of such malpractice. Pitiable, indeed, is the condition of the sick and the diseased in India. The council that deliberates for them, consists of the physician with his potions, the stupid barber and the Musselmen with their surgical case containing a razor and an awl, the exorcist drawing his circles and going through his list of trickeries, the astrologer with his book art of which he cites the prognosis that he pretends to have read among the stars; and the wretched patients are to be dosed with the compound tincture of quackery, witchcraft, and astrology prepared by this juggling college of physicians. I may well quote in this connection a proverb current in Hindoostan: "He who has killed off ten patients is a perfect doctor."

